# Utilization of a paediatric feeding tube for temporary arterial revascularisation/vascular salvage in traumatic extremity injury within a regional hospital setting

**DOI:** 10.1093/jscr/rjae509

**Published:** 2024-08-19

**Authors:** Johnny Arakkakunnel, Damian Fry

**Affiliations:** University of Southern Queensland, General Surgery, Toowoomba Base Hospital, Toowoomba, QLD 4350, Australia; General Surgery, Toowoomba Base Hospital, Toowoomba, QLD 4350, Australia

**Keywords:** temporary arterial revascularisation, trauma, vascular surgery

## Abstract

We present the management of a 16-year-old female patient with a complete transection of the right brachial artery, following a close-range high velocity penetrating gunshot wound. Due to the unique challenges posed in a regional setting, lack of formal vascular expertise on-site, and inability to transfer to a vascular trauma centre, a Temporary Intravascular Shunt was employed using a paediatric feeding tube to revascularise the limb. This article highlights the successful temporary revascularization approach and the importance of resourcefulness in managing complex vascular trauma in non-specialized centres.

## Introduction

Temporary intravascular shunts (TIVS) are essential for rapid haemorrhage control and restoring perfusion in vascular trauma before definitive repair. Despite increasing retrospective evidence supporting TIVS in trauma, clinical reports remain limited.

Studies highlight the importance of swift revascularisation for limb salvage in traumatic arterial injuries [1, 2]. In vascular injuries with limb fractures, prompt transport to the operating theatre for revascularisation is crucial for successful limb salvage in upper extremity arterial injuries [3, 4].

Vascular injuries, particularly in regional settings lacking immediate access to specialized vascular services, pose significant challenges. This report details the case of a young, healthy female who sustained a proximal arterial injury from a close-range shotgun wound. Limited resources and expertise in the regional centre necessitated a temporary revascularization approach to save the devascularized limb until definitive care was possible.

## Case report

A 16-year-old female with no significant medical history presented with a complete transection of the right brachial artery, an open comminuted humerus fracture, median nerve injury, and soft tissue shrapnel damage from a close-range high-velocity gunshot wound to the medial right upper limb. She arrived at a Level III trauma centre, which had immediate access to a 24-hour Emergency Department and General Surgery and Orthopaedics but lacked Trauma, Vascular, Plastics, and Interventional Radiology services.

A tourniquet was applied by ambulance services to control bleeding. On arrival, the patient received three units of packed red blood cells, tranexamic acid, fibrinogen, ADT, and intravenous antibiotics. She was haemodynamically stable but had lost about 1.5 l of blood. The right hand was cool, pulseless and did not bleed upon pinprick, indicating devascularization distal to the injury site. Clinical examination confirmed the need for revascularization, decided in consultation with a tertiary vascular service.

Adverse weather conditions and unavailability of transport made transfer to a Level I trauma centre unfeasible. Hence, a temporary intervention was imperative to prevent limb ischaemia and amputation. In consultation with the tertiary hospital’s trauma vascular team via telecommunication, a temporary intravascular shunt (TIVS) was placed in the emergency department resuscitation room by the general surgical and orthopaedic teams. The patient was intubated before the procedure.

## Surgical technique

**Figure 1:**
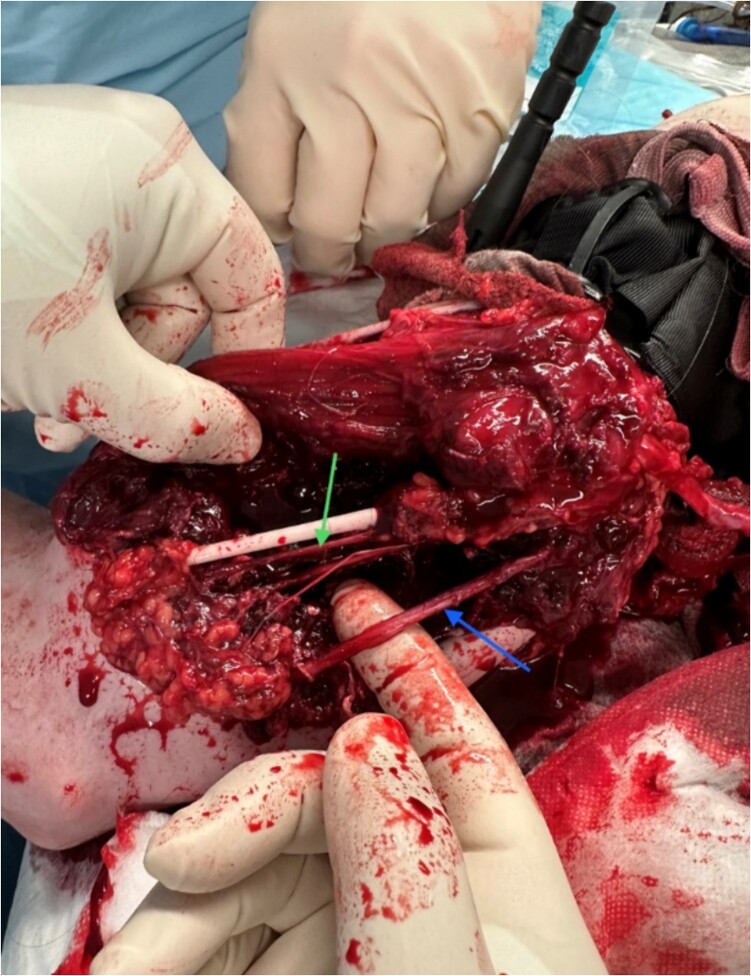
Identification of important structures and damage sustained; torniquet in situ; green arrow: medial brachial cutaneous nerve; blue arrow: ulnar nerve.

**Figure 2:**
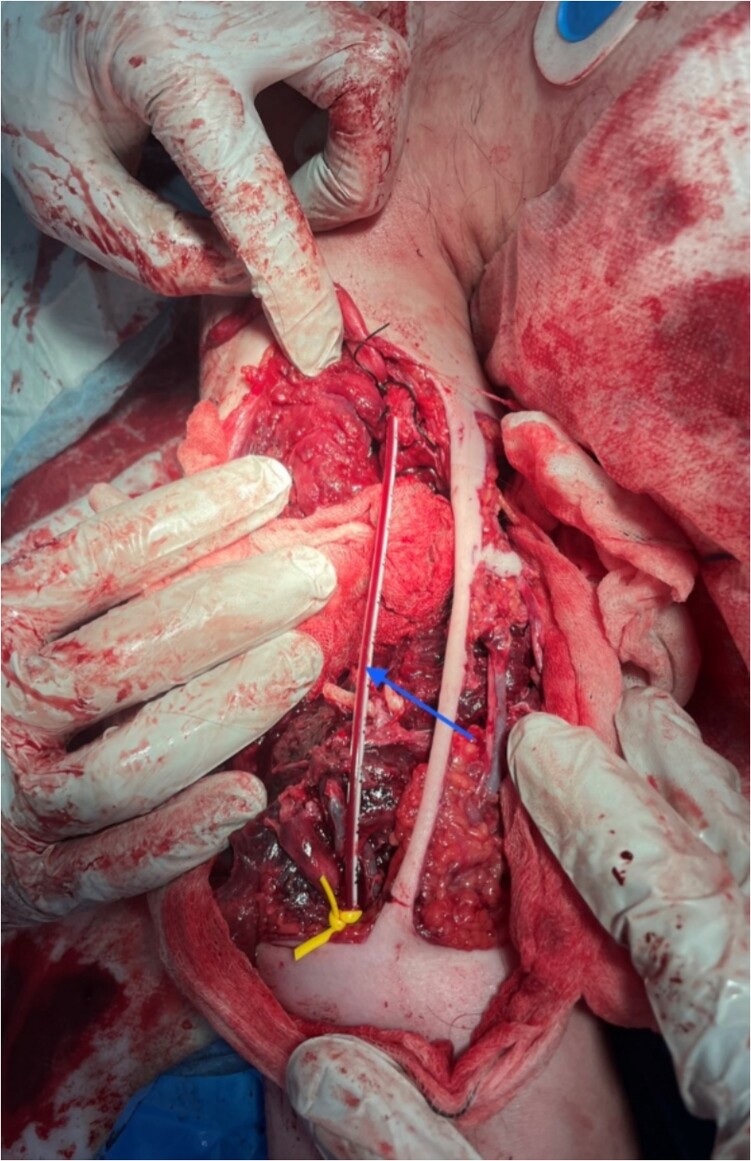
Insertion of paediatric feeding tube as TIVS w/ reperfusion; blue arrow: paediatric feeding tube tied to ends of transected brachial artery.

(i) Identification of anatomical structures and damage sustained (Fig. 1)
  (a) 10 cm brachial artery defect, no active bleeding.
  (b) Radial and ulnar nerves identified and preserved.
  (c) Medial nerve likely transected.
  (d) 3-4 cm humeral defect.
(ii) Tourniquet released.
(iii) Temporary clamping of distal and proximal ends of brachial artery.
(iv) Transverse arteriotomy with heparinization of the proximal and distal ends.
(v) Intraluminal arterial clot evacuation using a Fogarty catheter.
(vi) Insertion of a paediatric feeding tube into both ends of the brachial artery and fixation with sutures (Fig. 2).
(vii) Wound packing, application of a backslab for fracture stabilisation, and antibiotic coverage.
(viii) Commencement of a heparin infusion.

Immediate limb reperfusion was confirmed by colour and bleeding response to pinprick. The intervention successfully restored blood flow, enabling the patient’s transfer to a tertiary trauma centre for definitive care. She was closely monitored for reperfusion-related injuries throughout her treatment.

At the tertiary trauma centre, a multidisciplinary team of vascular, plastic surgery, and orthopaedic specialists managed the patient. Her injuries included

**Figure 3:**
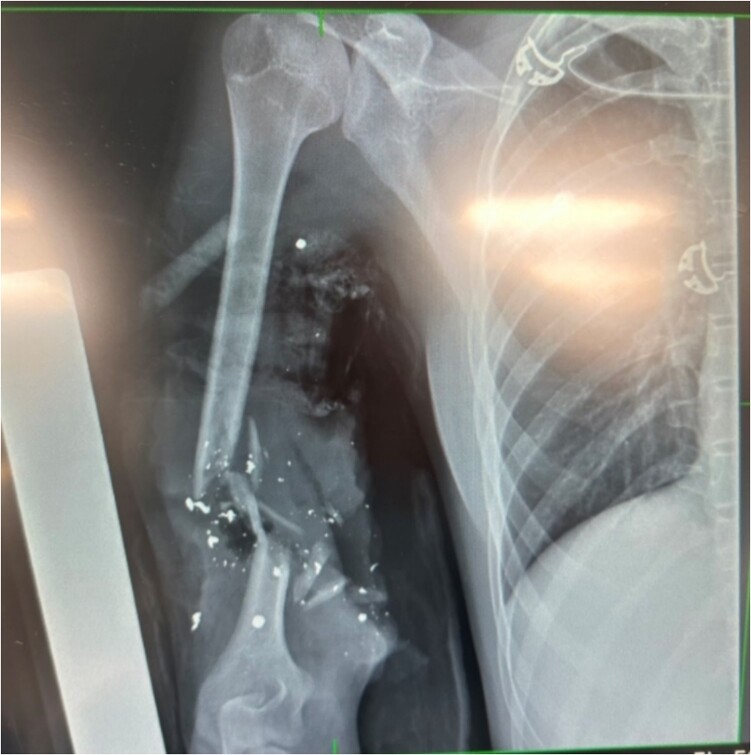
X-ray showing extent of damage to humerus; bullet shrapnel visible.

(i) Avulsed lateral cutaneous nerve of the forearm.
(ii) Avulsed medial cutaneous nerve of the arm.
(iii) Transected median nerve (permanent injury).
(iv) Muscle loss in the biceps and partial loss in the brachialis and triceps.
(v) Comminuted humeral fracture (Fig. 3).
(vi) Large soft tissue defect requiring plastic reconstruction with a distal free flap.

Initial management at the tertiary centre included open reduction and external fixation of the humerus, brachial-brachial artery bypass using a saphenous vein graft, necrotic tissue debridement, and a distal free flap procedure.

During recovery, the patient experienced ongoing neurological deficits, which improved with rehabilitation. She now has a well-perfused limb without distal tissue loss, and amputation was avoided.

## Discussion

This case highlights the successful use of an unconventional yet effective technique for temporary vascular shunting in a challenging clinical scenario. Despite lacking on-site vascular expertise and the inability to promptly transfer the patient, the urgent need for revascularization was recognized. Guided by a tertiary care vascular team, the surgical team resourcefully utilized a paediatric feeding tube as an improvised TIVS, avoiding major morbidity and salvaging the ischaemic limb before definitive repair.

This case underscores the importance of timely vascular intervention and the necessity of creative solutions when resources are scarce. Extremity vascular trauma requires rapid haemorrhage control and perfusion restoration to prevent limb loss, as delays beyond 6 hours significantly increase morbidity [1, 5]. Early shunting is vital when definitive repair is not immediately feasible.

The use of a paediatric feeding tube for temporary arterial shunting in peripheral vascular trauma proved effective. While silastic or intraluminal vascular shunts are typical, prior reports have described using feeding tubes, chest tubes, and sterile plastic tubing for damage control [6, 7]. Our experience aligns with these reports, demonstrating adequate flow restoration until vein grafting was possible.

This case exemplifies the value of improvisation and multidisciplinary collaboration in managing complex trauma in regional settings with limited resources. Despite significant challenges, optimal patient outcomes were achieved through close coordination across specialties and resourceful thinking, guided by the tertiary vascular team via telecommunication.

In the era of damage control surgery, this case suggests that limb salvage is possible even without on-site specialized vascular capabilities, given proper collaboration, resourcefulness, and a damage control mindset. Our experience adds to the growing literature on innovative TIVS techniques for vascular trauma management, highlighting the importance of timely shunting, improvisation, and coordination between centres in rural locations.

## Conclusion

Temporary revascularization using paediatric feeding tubes can effectively manage proximal arterial injuries in regional settings with limited vascular expertise. Early collaboration with orthopaedic and vascular teams is essential to optimize outcomes in such cases.
